# Supplementary material for: A Diet Enriched with Lacticaseibacillus rhamnosus HN001 and Milk Fat Globule Membrane Alters the Gut Microbiota and Decreases Amygdala GABA a Receptor Expression in Stress-Sensitive Rats
Source: Int J Mol Sci. 2023 Jun 21;24(13):10433. doi: 10.3390/ijms241310433 (PMC10341670; doi:10.3390/ijms241310433)
Supplement: Supplementary file 1 [file ijms-24-10433-s001.zip › ijms-2446160-supplementary.pdf]

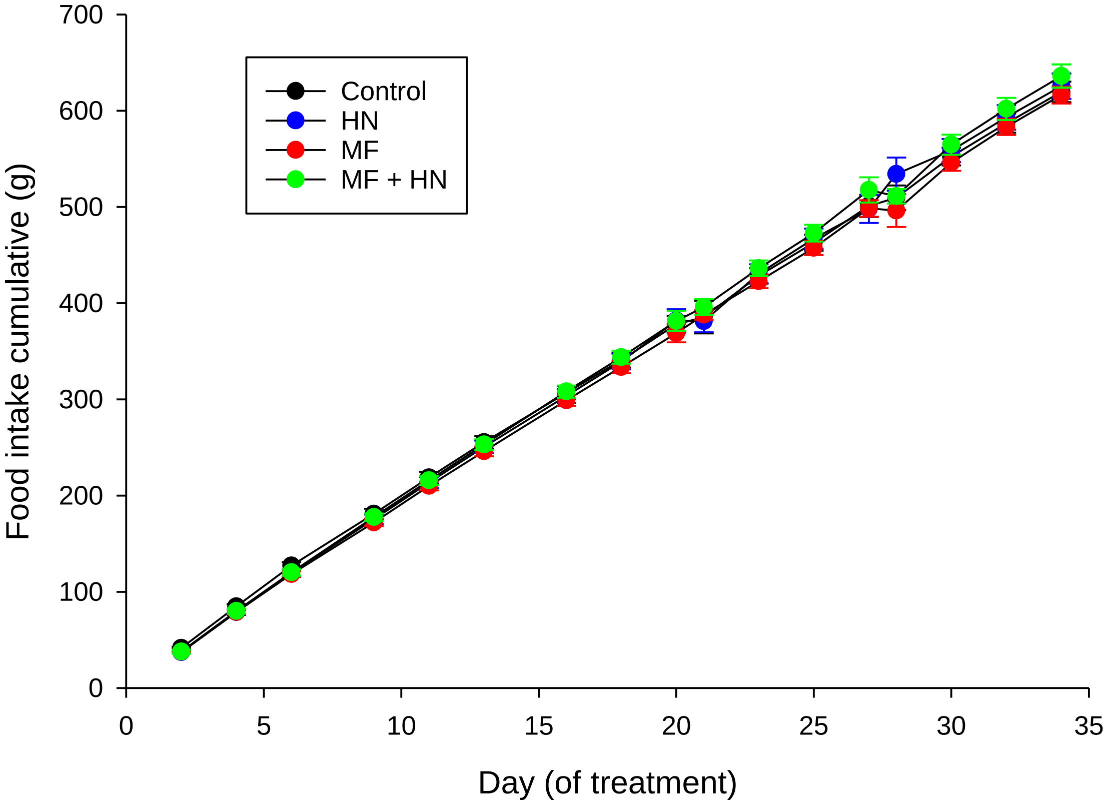

Supplementary Figure S1. Food intake over the duration of the study for: HN; *Lacticaseibacillus rhamnosus* strain HN001 (LactoB HN001™), MF; milk fat globule membrane (Surestart™ MFGM Lipid 70).

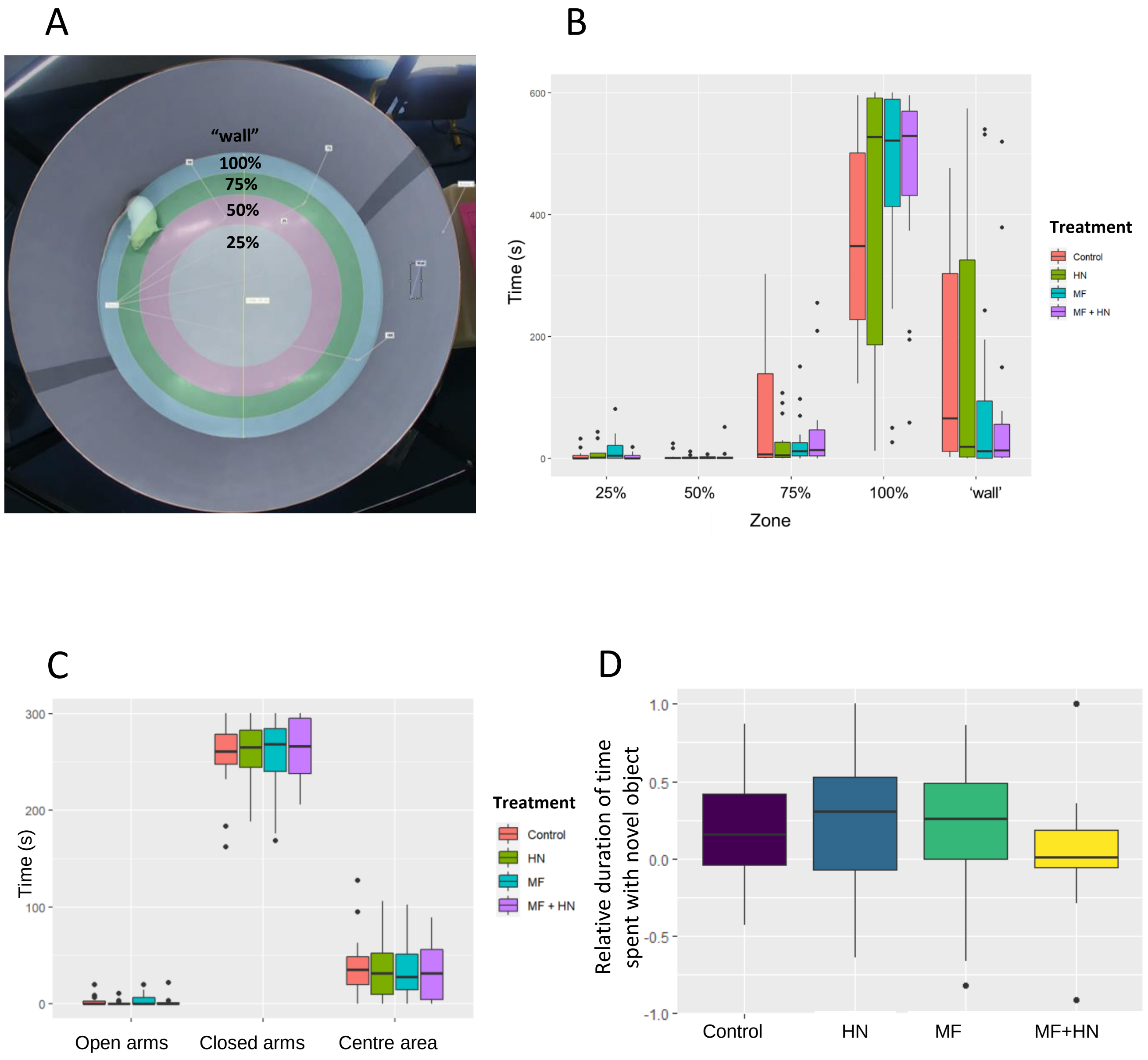

Supplementary Figure S2. (A) The open field test arena is divided into zones from the center 25% (white), 50% (pink), 75% (green), 100% (blue), to the wall (grey) and (B) shows corresponding results for time spent in each zone for each treatment group. Rats fed treatment diets did not show any difference in velocity, or distance travelled, entry to the inner zone of the arena or time spent in the inner zone compared with control animals. The PERMANOVA also found no evidence of an impact of diet or pre-study weight on the behaviour exhibited by the animals in the test. (C) In the elevated plus maze, rats fed treatment diets did not spend any more time in the open arms than control rats.
(D) In the novel object recognition test rats fed treatment diets did not spend any more time than control animals exploring the novel versus the familiar object (relative duration = time spent with the novel object minus the time spent with the familiar object divided by the total time for both objects). HN; *Lacticaseibacillus rhamnosus* strain HN001 (LactoB HN001™), MF; milk fat globule membrane (Surestart™ MFGM Lipid 70).

Accession#	Gene name	Probe function
ERCC_00126.1:220	NEG_F (0) **	Negative
ERCC_00098.1:785	NEG_E (0) **	Negative
ERCC_00019.1:140	NEG_C (0) **	Negative
ERCC_00096.1:230	NEG_A (0) **	Negative
ERCC_00041.1:440	NEG_B (0) **	Negative
ERCC_00076.1:355	NEG_D (0) **	Negative
ERCC_00034.1:195	POS_A (128) **	Positive
ERCC_00092.1:540	POS_D (2) **	Positive
ERCC_00117.1:385	POS_F (0.125) **	Positive
ERCC_00002.1:850	POS_C (8) **	Positive
ERCC_00112.1:695	POS_B (32) **	Positive
ERCC_00035.1:485	POS_E (0.5) **	Positive
NM_013034.3:586	Slc6a4 (5HTT) *	reference
NM_001109883.2:880	Abcf1	reference
NM_017015.2:1730	Gusb	reference
NM_017025.1:1360	Ldha	reference
NM_031773.1:2785	Polr1b	reference
NM_022402.2:1	Rplp0	reference
NM_030991.1:725	Snap25	reference
NM_053788.2:1620	Stx1a	reference
NM_053859.1:790	Slc17a7	reference
NM_012583.2:20	Hprt	reference
NM_031003.2:382	Abat	
NM_022193.1:2518	Acaca	
NM_012739.2:1155	Adra2a	
NM_012513.4:637	Bdnf	
NM_031561.2:1470	Cd36	
NM_012531.2:1070	Comt	
NM_001034925.2:2264	Cpt1c	
NM_001007703.1:1810	Cptp	
NM_031019.1:625	crf	
NM_012546.2:1077	Drd1	
NM_012547.1:2353	Drd2	
NM_012768.1:310	Drd5	
NM_001191796.1:478	Elovl4	
NM_134383.2:266	Elovl6	
NM_172036.2:180	Gabarap	
NM_031028.3:1225	Gabbr1	
NM_031802.1:590	Gabbr2	
NM_183326.2:1157	Gabra1	
NM_001135779.1:1130	Gabra2	
NM_080587.3:715	Gabra4	
NM_017295.1:842	Gabra5	
NM_021841.1:1050	Gabra6	
NM_012956.1:750	Gabrb1	
NM_012957.2:800	Gabrb2	
NM_017289.1:410	Gabrd	
NM_023091.1:2550	Gabre	
NM_080586.1:1244	Gabrg1	
NM_183327.1:670	Gabrg2	
NM_017007.1:260	Gad1	
NM_012563.1:585	Gad2	
NM_017335.1:1950	Slc6a1 (Gat1) *	
NM_133623.1:1508	Slc6a13 (Gat2) *	
NM_024372.1:1390	Slc6a11 (Gat3) *	
NM_012569.2:982	Gls	

Accession#	Gene name
NM_001035233.1:1198	Slc1a2 (Glt-.1) *
NM_012570.1:1370	Glud1
NM_012767.2:326	Gnrh1
NM_031608.1:855	Gria1
NM_017261.2:1532	Gria2
NM_001112742.1:1246	Gria3
NM_133560.2:1066	Trak2 (GRIF-1) *
NM_017241.2:615	Grik1
NM_019309.2:2450	Grik2
NM_012572.1:1625	Grik4
NM_031508.2:1060	Grik5
NM_017010.1:2615	Grin1
NM_012573.2:3365	Grin2a
NM_012574.1:3170	Grin2b
NM_012575.3:2348	Grin2c
NM_022797.1:3296	Grin2d
NM_001114330.1:1897	Grm1
NM_001105712.1:2575	Grm3
NM_022666.1:2161	Grm4
NM_017012.1:2135	Grm5
NM_031040.1:1435	Grm7
NM_022202.1:1960	Grm8
NM_012585.1:600	Htr1a
NM_022225.1:615	Htr1b
NM_012852.1:560	Htr1d
NM_021857.1:215	Htr1f
NM_017254.1:440	Htr2a
NM_012765.3:789	Htr2c
NM_024394.2:1030	Htr3a
NM_012853.1:20	Htr4
NM_022938.2:520	Htr7
NM_012854.2:185	Il10
NM_022611.1:364	Il12b
NM_053828.1:200	Il13
NM_001106897.1:615	Il17a
NM_019165.1:110	Il18
NM_017019.1:580	Il1a
NM_031512.1:440	Il1r1
NM_053836.1:5	Il2
NM_012589.1:55	Il6
XM_342346.3:3560	Nfkb1
NM_052799.1:56	Nos1
NM_019256.1:2815	P2rx7
NM_001002853.1:170	P2ry13
NM_001077641.1:2579	Plcb1
NM_013196.1:1010	Ppara
NM_013141.2:1186	Pparg
NM_181386.2:1195	Sgms1
NM_022851.1:974	Aldh5a1
NM_012675.2:305	Tnf (tnfa) *
NM_001100634.2:2202	Tph1
NM_173839.2:1580	Tph2

Supplementary Table S1. NanoString nCounter Codeset probe list for brain tissue gene expression. The Codeset has a custom probe design of 12 control, 10 reference and 87 target genes. They are listed by RefSeq Accession numbers, Probeset ID (Official Gene names) and probe function.
( )*Trial name of gene that differs to Official Gene Name
( )**Concentration of positive and negative control probes

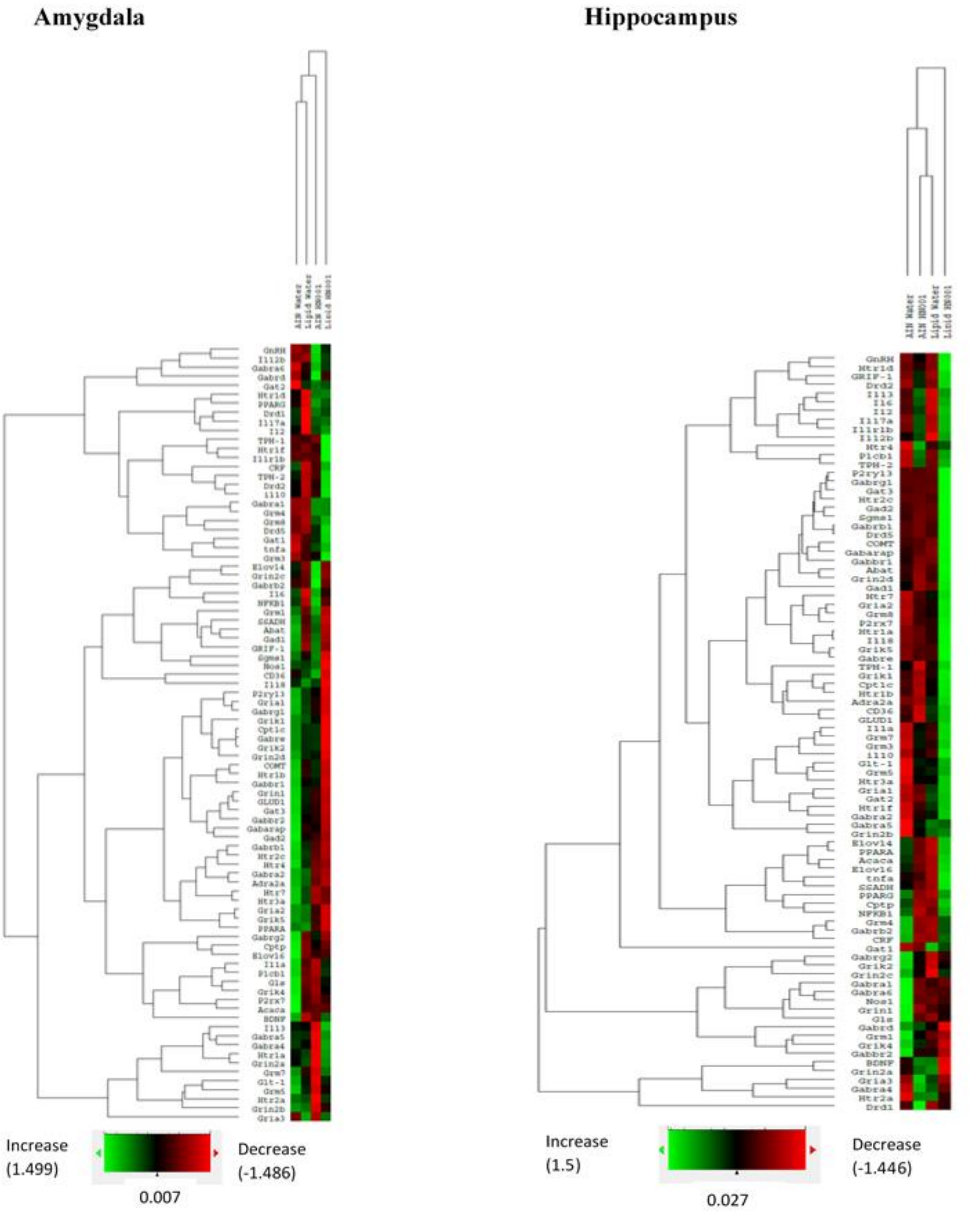

Supplementary Figure S3. Heatmaps of gene expression in the Amygdala and Hippocampus for the four treatment groups: AIN water (control), AIN HN001, Lipid water, Lipid HN001, by the 86 genes in the custom panel (excl. housekeeping genes). The Z-score transformation is by "Genes" with sample counts averaged for each treatment group using "Euclidean Distance" as the distance matrix. Increased expression is shown as "green", and decreased expression is shown as "red". HN001; *Lacticaseibacillus rhamnosus* strain HN001 (LactoB HN001™), Lipid; milk fat globule membrane (Surestart™ MFGM Lipid 70).

Tissue	Treatment	Gene	log2(FC)	FDR	Expression
Amy	AIN HN001	Grm4	1.05	0.0394	increased
Amy	Lipid HN001	Gabre	-2.11	0.0163	decreased
Amy	Lipid HN001	Gat3	-0.73	0.0163	decreased
Amy	Lipid HN001	Gabrg1	-0.418	0.043	decreased
Hip	Lipid HN001	Gabrd	-1.13	0.0357	decreased
Amy	Lipid HN001	*Gria1	-0.457	0.053	decreased
Amy	Lipid HN001	*Gabrb1	-0.394	0.0547	decreased
Amy	Lipid HN001	*Htr4	-0.733	0.0674	decreased
Amy	Lipid HN001	*Rplp0	-0.234	0.0674	decreased
Amy	Lipid HN001	*GLUD1	-0.363	0.0674	decreased
Amy	Lipid HN001	*Gabbr1	-0.286	0.0674	decreased
Amy	Lipid HN001	*Gabra2	-0.433	0.0674	decreased
Amy	Lipid HN001	*Grik2	-0.321	0.074	decreased
Amy	Lipid HN001	*Grik1	-0.348	0.074	decreased
Amy	Lipid HN001	*COMT	-0.243	0.0759	decreased
Amy	Lipid HN001	*Nos1	-0.41	0.0857	decreased
Amy	Lipid HN001	*Htr2c	-0.619	0.0857	decreased
Amy	Lipid HN001	*Grm3	0.216	0.0997	increased

Supplementary Table S2. Differential expression analysis using a lm.nb (log-linear negative binomial model) within the nSolver software. Significance values (FDR < 0.05) and log2fold changes (> 0.263) are relative to control group (AIN diet and water). *Grm4* (metabotropic glutamate receptor), *Gabre* (GABA A receptor epsilon subunit), *Gat3* (brain-specific GABA transporter), *Gabrg1* (GABA A receptor gamma 1 subunit), *Gabrd* (GABA A receptor delta subunit).
Genes with FDR values of > 0.05 < 0.1 show trends towards altered expression in the Amygdala. HN001; *Lacticaseibacillus rhamnosus* strain HN001 (LactoB HN001™), Lipid; milk fat globule membrane (Surestart™ MFGM Lipid 70).

Level 1	Level 2	Level 3	Level 4	logFC	FDR
Cell Envelope	Cell Envelope, Capsule and Slime layer	Capsule and Slime layer	Alginate biosynthesis	-0.4	0.0192
Cell Envelope	Cell Envelope, Capsule and Slime layer	Capsule and Slime layer	Lipid-linked oligosaccharide synthesis related cluster	0.52	0.034
Cell Envelope	Cell Envelope, Capsule and Slime layer	Cell wall of Mycobacteria	Mycobacterial cell wall virulence lipid phthiocerol dimycocerosate (PDIM)	-1.22	0.0068
Cell Envelope	Cell Envelope, Capsule and Slime layer	Gram-Positive (Monoderm) cell wall components	D-alanylation of teichoic acid	0.82	0.0007
Cellular Processes	Microbial communities	Quorum sensing and biofilm formation	Autoinducer 2 (AI-2) transport and processing (lsrACDBFGE operon)	0.39	0.0025
Cellular Processes	Prokaryotic cell type differentiation	Sporulation	Spore germinant receptors	0.4	0.0107
Energy	Energy and Precursor Metabolites Generation	Central Metabolism	Butyrate kinase pathway	-0.39	0.0006
Energy	Energy and Precursor Metabolites Generation	Central Metabolism	Dihydroxyacetone kinases	0.29	0.0234
Energy	Energy and Precursor Metabolites Generation	Central Metabolism	Pyruvate formate-lyase cluster	0.3	0.0272
Energy	Respiration	Electron donating reactions	Energy-conserving hydrogenase (ferredoxin)	-0.95	0.0001
Metabolism	Amino Acids and Derivatives	Arginine_ urea cycle, creatine, polyamines	Anaerobic Oxidative Degradation of L-Ornithine	0.32	0.0022
Metabolism	Amino Acids and Derivatives	Arginine_ urea cycle, creatine, polyamines	Cyanophycin Metabolism	0.31	0.0263
Metabolism	Amino Acids and Derivatives	Arginine_ urea cycle, creatine, polyamines	Methylhydantoinase	0.5	0.0048
Metabolism	Amino Acids and Derivatives	Lysine, threonine, methionine, and cysteine	Fructoselysine and glucoselysine	1.03	>0.0001
Metabolism	Amino Acids and Derivatives	Lysine, threonine, methionine, and cysteine	Lysine fermentation to crotonoyl-CoA	0.54	>0.0001
Metabolism	Amino Acids and Derivatives	Lysine, threonine, methionine, and cysteine	S-methylmethionine	1.21	0.0032
Metabolism	Carbohydrates	Amino sugars and nucleotide sugars	Hyaluronate utilization	0.29	0.0001
Metabolism	Carbohydrates	C-1 compound metabolism	Formaldehyde assimilation: Ribulose monophosphate pathway	0.62	0.0003
Metabolism	Carbohydrates	Carboxylic acids	Alpha-acetolactate operon	0.95	0.0116
Metabolism	Carbohydrates	Monosaccharides	L-ascorbate utilization (and related gene clusters)	0.54	0.012
Metabolism	Carbohydrates	Sugar alcohols	Propanediol utilization	0.39	0.0001
Metabolism	Cofactors, Vitamins, Prosthetic Groups	Folate and pterines	p-Aminobenzoyl-Glutamate Utilization	0.44	>0.0001
Metabolism	Cofactors, Vitamins, Prosthetic Groups	Quinone cofactors	Menaquinone biosynthesis from chorismate via 1,4-dihydroxy-6-naphthoate	-0.35	0.0051
Metabolism	Cofactors, Vitamins, Prosthetic Groups	Quinone cofactors	Pyrroloquinoline Quinone biosynthesis	0.65	0.0007
Metabolism	Cofactors, Vitamins, Prosthetic Groups	Thiamin	Biosynthesis of thiamin antivitamin Bacimethrin	0.51	0.0099

Level 1	Level 2	Level 3	Level 4	logFC	FDR
Metabolism	Cofactors, Vitamins, Prosthetic Groups	Quinone cofactors	Pyrroloquinoline Quinone biosynthesis	0.65	0.0007
Metabolism	Cofactors, Vitamins, Prosthetic Groups	Thiamin	Biosynthesis of thiamin antivitamin Bacimethrin	0.51	0.0099
Metabolism	Cofactors, Vitamins, Prosthetic Groups	Thiamin	Thiamin, hydroxymethylpyrimidine selected transporters	0.81	0.0075
Metabolism	Fatty Acids, Lipids, and Isoprenoids	Fatty acids	Enoyl-[ACP] reductases disambiguation	0.53	0.0494
Metabolism	Fatty Acids, Lipids, and Isoprenoids	Isoprenoids	Acyclic terpene utilization	-1.12	0.027
Metabolism	Fatty Acids, Lipids, and Isoprenoids	Isoprenoids	Mevalonate metabolic pathway	0.96	0.0081
Metabolism	Iron acquisition and metabolism	Siderophores	Siderophore Mycobactin	-0.92	0.0005
Metabolism	Iron acquisition and metabolism	Siderophores	Siderophore Pyoverdine	-0.96	0.0016
Metabolism	Nucleosides and Nucleotides	Purines	Purine catabolism in Bacillus subtilis	0.28	0.0022
Metabolism	Nucleosides and Nucleotides	Purines	Xanthine dehydrogenase subunits	0.35	>0.0001
Miscellaneous	Prophages, Transposable elements, Plasmids	Phages, Prophages	P2-like phage	-0.36	0.0426
Protein Processing	Protein Fate (folding, modification, targeting, degradation)	Protein glycosylation in Prokaryotes	N,N'-diacetylbacillosamine	-0.54	0.007
Protein Processing	Protein Fate (folding, modification, targeting, degradation)	Protein targeting, sorting, translocation	SecY2-SecA2 Specialized Transport System	0.61	0.0022
Protein Processing	Protein Fate (folding, modification, targeting, degradation)	Selenoproteins	Glycine reductase, sarcosine reductase and betaine reductase	0.49	>0.0001
Protein Processing	Protein Fate (folding, modification, targeting, degradation)	Selenoproteins	Selenocysteine metabolism	0.48	>0.0001
Protein Processing	Protein Synthesis	Aminoacyl-tRNA-synthetases	tRNA aminoacylation, Pyr	0.83	0.0065
Stress Response, Defense, Virulence	Stress Response, Defense and Virulence	Resistance to antibiotics and toxic compounds	Aminoglycoside modifying enzymes: O-phosphotransferases	-0.68	>0.0001
Stress Response, Defense, Virulence	Stress Response, Defense and Virulence	Resistance to antibiotics and toxic compounds	Beta-lactamases Ambler class B	0.47	0.0008
Stress Response, Defense, Virulence	Stress Response, Defense and Virulence	Resistance to antibiotics and toxic compounds	Macrolides, lincosamides, streptogramins, ketolides, oxazolidinones (MLSKO) resistance: rRNA methylases	-1.05	>0.0001

Supplementary Table S3. Functional genes annotated to the SEED database at level 4 with significant differential abundances (FDR<0.05, |logFC| > 0.263) between rats fed the control non-Lipid 70 diet or Lipid 70 (Surestart™ MFGM Lipid 70). Positive logFC value indicates higher abundance in Lipid 70 group, while negative logFC value indicates higher abundance in the non-Lipid 70 group.

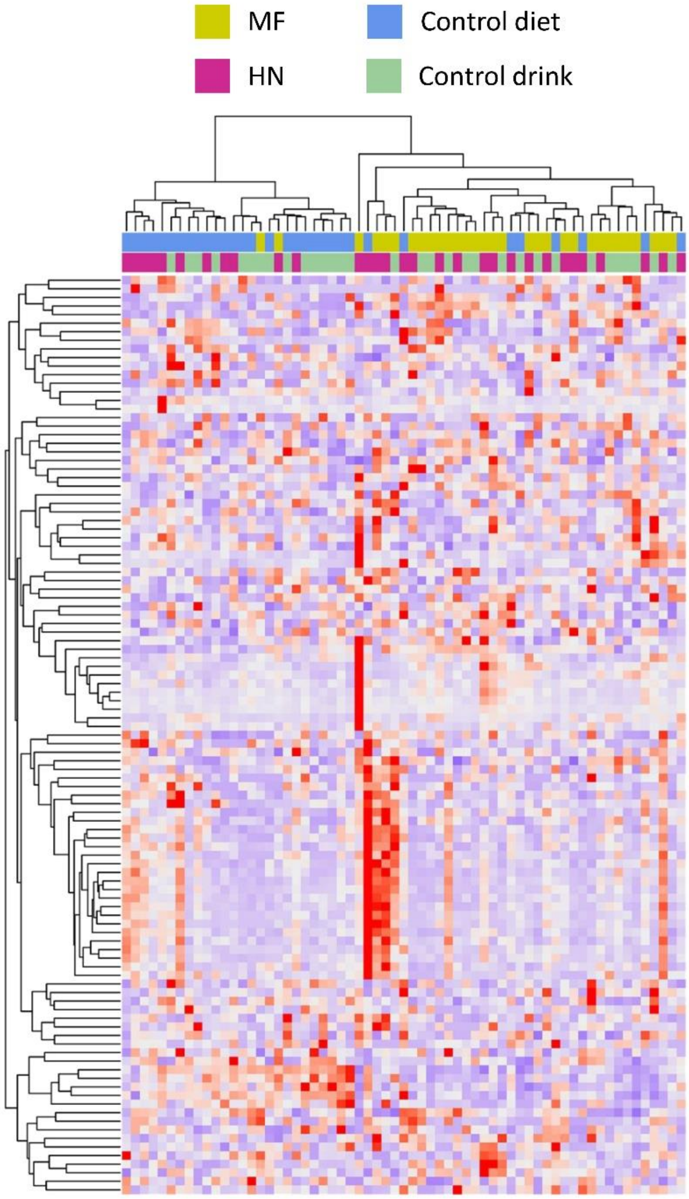

Supplementary Figure S4. Heatmap showing hierarchical clustering of SEED level 4 function profiles in the caecal metagenome for functions that exhibit the greatest variation (top 5% coefficient of variation) in abundance across all rats, regardless of group. SEED functions in rows and rats represented by each column. Colour bar across top indicates treatment group for each rat, showing whether they received the control AIN diet (blue), MF diet (yellow), plain drinking water control (green) or HN probiotic in drinking water (magenta). HN; *Lacticaseibacillus rhamnosus* strain HN001 (LactoB HN001™), MF; milk fat globule membrane (Surestart™ MFGM Lipid 70).

A

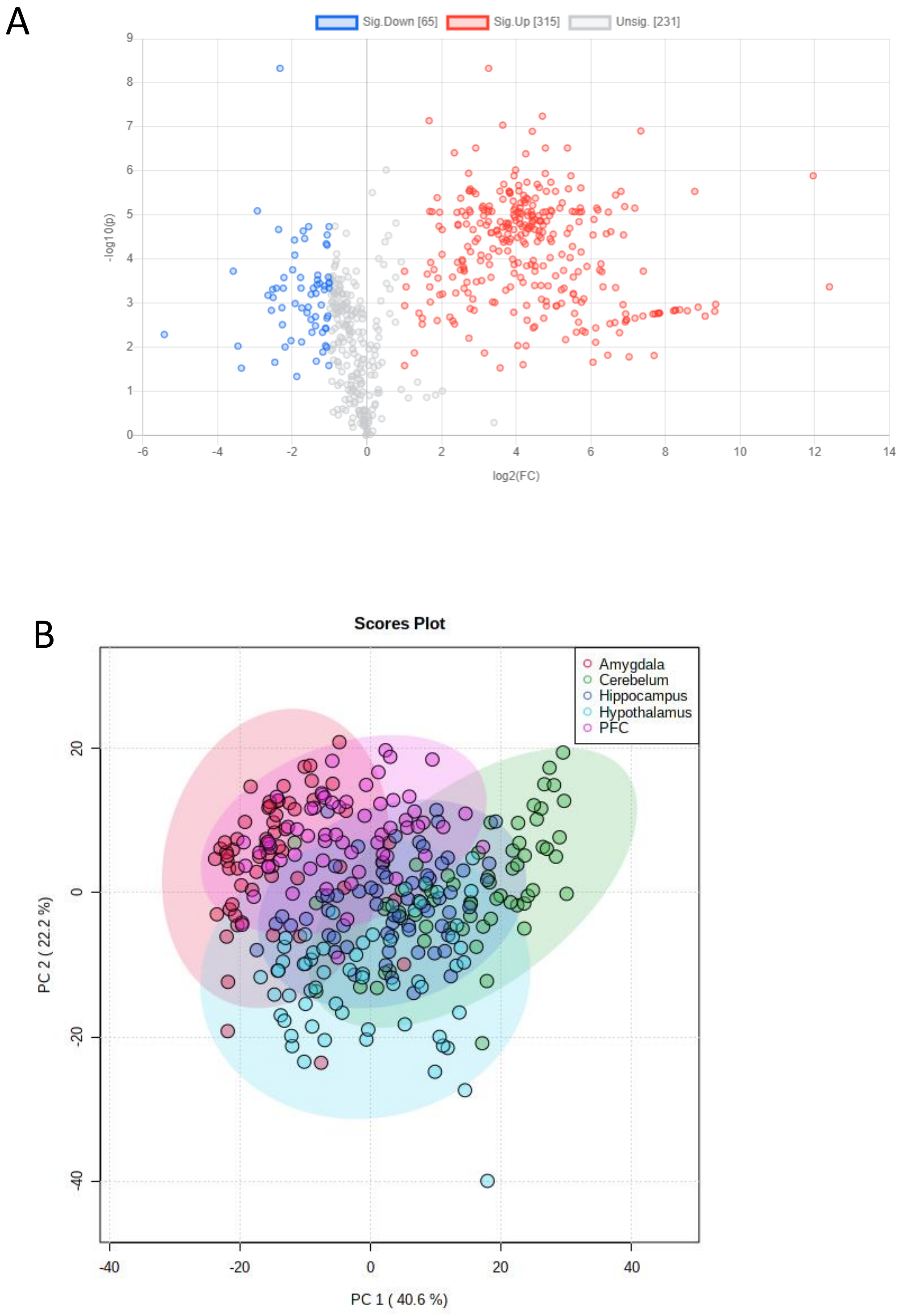

B

Supplementary Figure S5. (A) Volcano plot of lipids significantly differentiating (FDR p-value <0.05, Fold-change>2) between the two diets with 315 lipids higher in the MF and 65 lower in the MF compared to the control diet. (B) Principal component analysis score plot of brain lipids detected across the 5 brain regions. MF; milk fat globule membrane (Surestart^{TM} MFGM Lipid 70).

A)

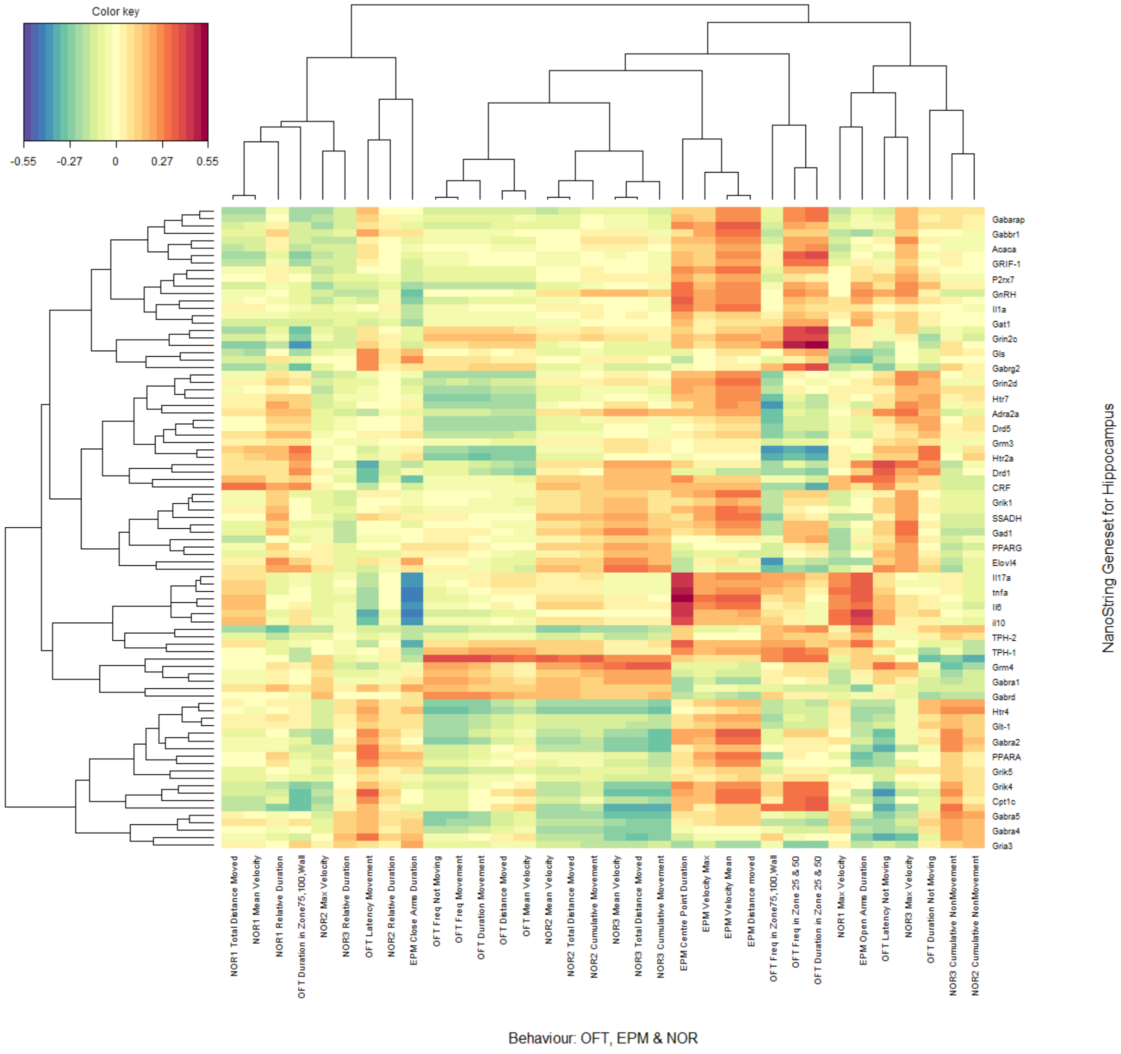

B)

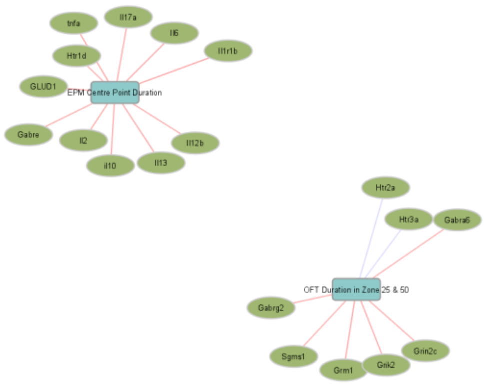

Supplementary Figure S6. Correlations obtained with sPLS – canonical on selected Behaviour measurements and gene expression counts in the Hippocampus. Heatmap (A) indicating hierarchical clustering of correlations between subsets of variables are described in the “Colour Key”, with positive (red), negative (blue) and weak shown as lighter colours. Relevance networks show the pair-wise similarity matrix of correlated positive (red) and negative (blue) variables. Behaviour and gene expression variables are represented as rectangles and ellipses respectively.

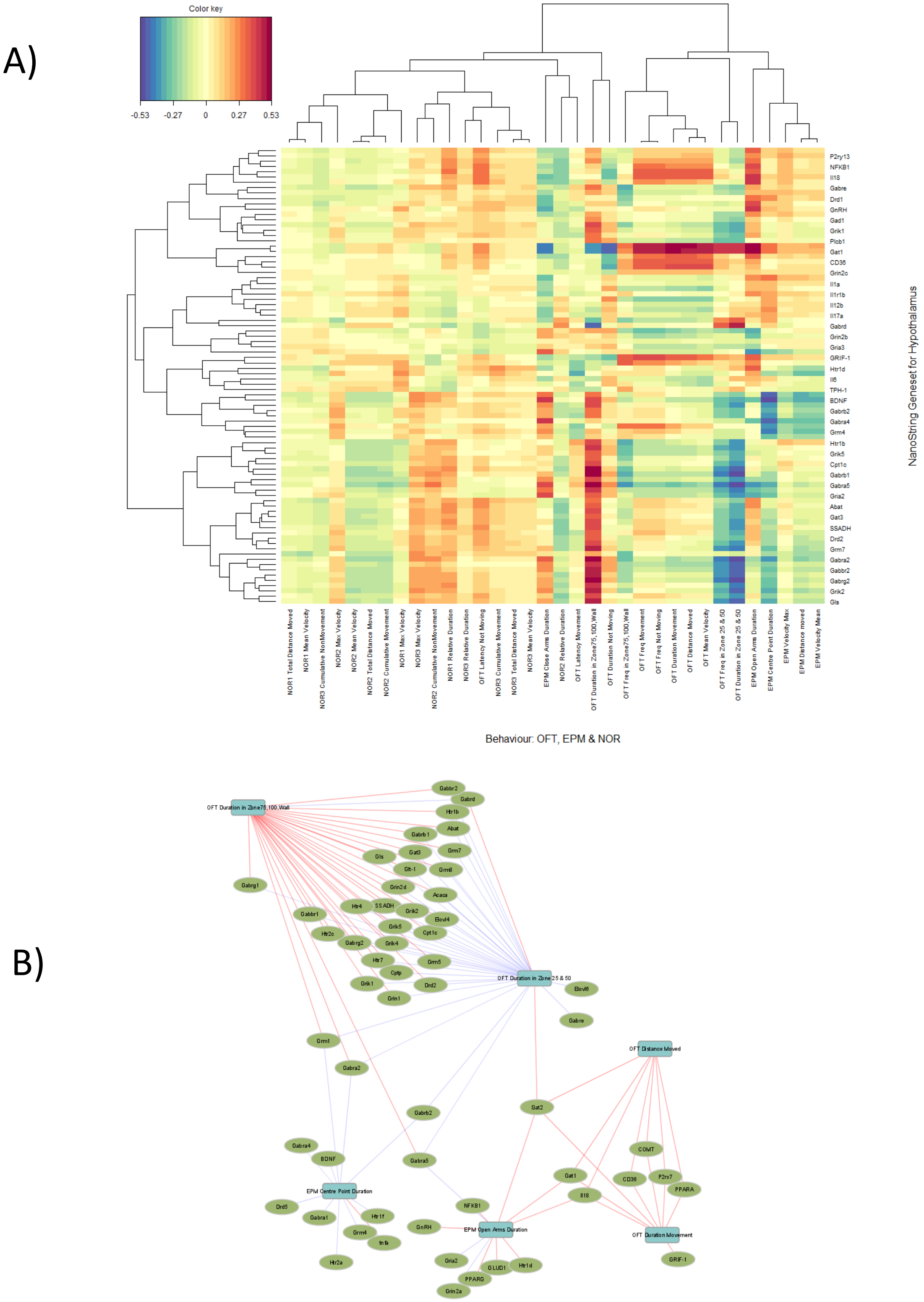

Supplementary Figure S7. Correlations obtained with sPLS – canonical on selected Behaviour measurements and gene expression counts in the Hypothalamus. Heatmap (A) indicating hierarchical clustering of correlations between subsets of variables are described in the “Colour Key”, with positive (red), negative (blue) and weak shown as lighter colours. Relevance networks show the pair-wise similarity matrix of correlated positive (red) and negative (blue) variables. Behaviour and gene expression variables are represented as rectangles and ellipses respectively.

A)

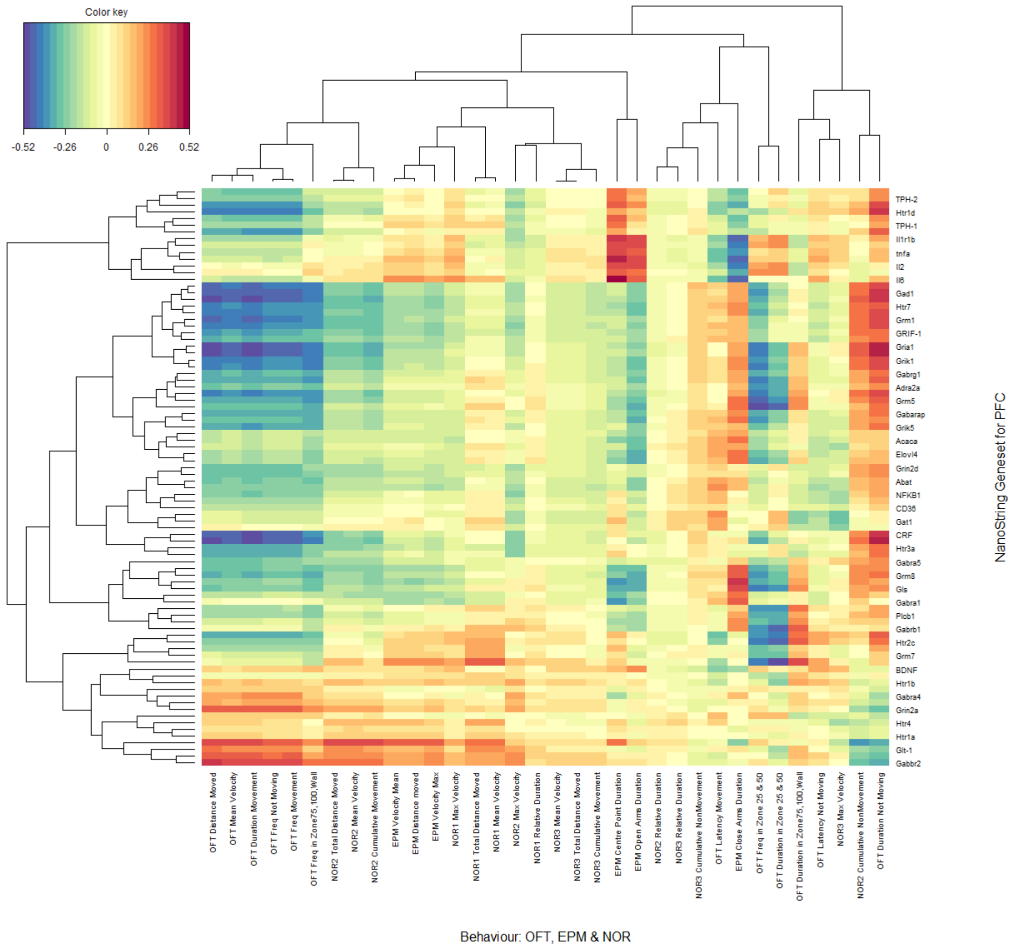

B)

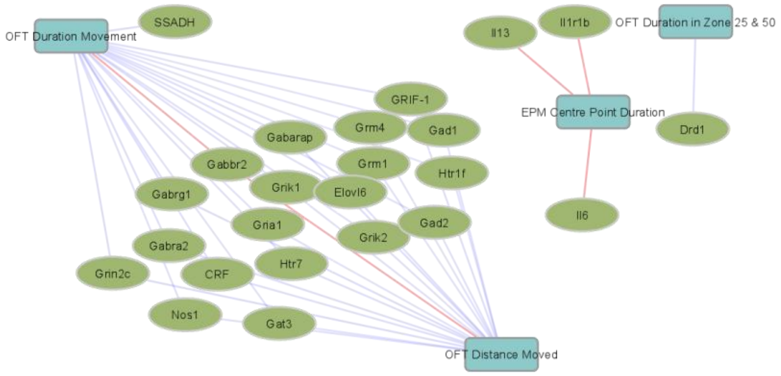

Supplementary Figure S8. Correlations obtained with sPLS – canonical on selected Behaviour measurements and gene expression counts in the PFC. Heatmap (A) indicating hierarchical clustering of correlations between subsets of variables are described in the "Colour Key", with positive (red), negative (blue) and weak shown as lighter colours. Relevance networks show the pair-wise similarity matrix of correlated positive (red) and negative (blue)
variables. Behaviour and gene expression variables are represented as rectangles and ellipses respectively.
